# SLI-1 Cbl Inhibits the Engulfment of Apoptotic Cells in *C. elegans* through a Ligase-Independent Function

**DOI:** 10.1371/journal.pgen.1003115

**Published:** 2012-12-13

**Authors:** Courtney Anderson, Shan Zhou, Emma Sawin, H. Robert Horvitz, Michael E. Hurwitz

**Affiliations:** 1 Yale Cancer Center and Department of Medicine, Yale University School of Medicine, New Haven, Connecticut, United States of America; 2 Howard Hughes Medical Institute (HHMI), Department of Biology, Massachusetts Institute of Technology, Cambridge, Massachusetts, United States of America; University of California San Francisco, United States of America

## Abstract

The engulfment of apoptotic cells is required for normal metazoan development and tissue remodeling. In *Caenorhabditis elegans*, two parallel and partially redundant conserved pathways act in cell-corpse engulfment. One pathway, which includes the small GTPase CED-10 Rac and the cytoskeletal regulator ABI-1, acts to rearrange the cytoskeleton of the engulfing cell. The CED-10 Rac pathway is also required for proper migration of the distal tip cells (DTCs) during the development of the *C. elegans* gonad. The second pathway includes the receptor tyrosine kinase CED-1 and might recruit membranes to extend the surface of the engulfing cell. Cbl, the mammalian homolog of the *C. elegans* E3 ubiquitin ligase and adaptor protein SLI-1, interacts with Rac and Abi2 and modulates the actin cytoskeleton, suggesting it might act in engulfment. Our genetic studies indicate that SLI-1 inhibits apoptotic cell engulfment and DTC migration independently of the CED-10 Rac and CED-1 pathways. We found that the RING finger domain of SLI-1 is not essential to rescue the effects of SLI-1 deletion on cell migration, suggesting that its role in this process is ubiquitin ligase-independent. We propose that SLI-1 opposes the engulfment of apoptotic cells via a previously unidentified pathway.

## Author Summary

Cell death is a normal part of organismal development. When cells die, other cells engulf them. In the roundworm *C. elegans*, engulfment is facilitated by one pathway that rearranges the actin cytoskeleton and another that recruits membrane. Together they cause the formation of cellular extensions that surround the dead cell. Notably, little is known about how engulfment is inhibited. The cytoskeletal regulatory pathway, which also promotes cell migration, includes CED-10 and ABI-1, homologs of the actin regulators Rac and the Abi proteins, respectively. In mammals, the c-Cbl proto-oncogene interacts with Rac and Abi2 and has been shown to regulate the actin cytoskeleton, so we tested whether the *C. elegans* homolog of Cbl, SLI-1, regulates engulfment and cell migration. We found that SLI-1 inhibits both processes. Our analysis further showed that SLI-1 does not function by inhibiting other known engulfment proteins. Cbl proteins have ubiquitin ligase domains through which they target proteins for destruction or sequestration. Most of the known functions of Cbl proteins require that domain, but we found that SLI-1 did not require it to block engulfment and cell migration. We propose that SLI-1 inhibits engulfment and cell migration through a previously unidentified pathway.

## Introduction

The engulfment of apoptotic cells requires at least two processes to occur in the engulfing cell at the interface with the dying cell. Actin cytoskeletal elements need to be reorganized and membrane needs to be recruited. Together, these two processes result in the engulfing cell surrounding the dying cell. Two conserved molecular pathways were originally identified in *Caenorhabditis elegans* that are required for apoptotic cell engulfment and regulate these two processes.

**Figure 1:**
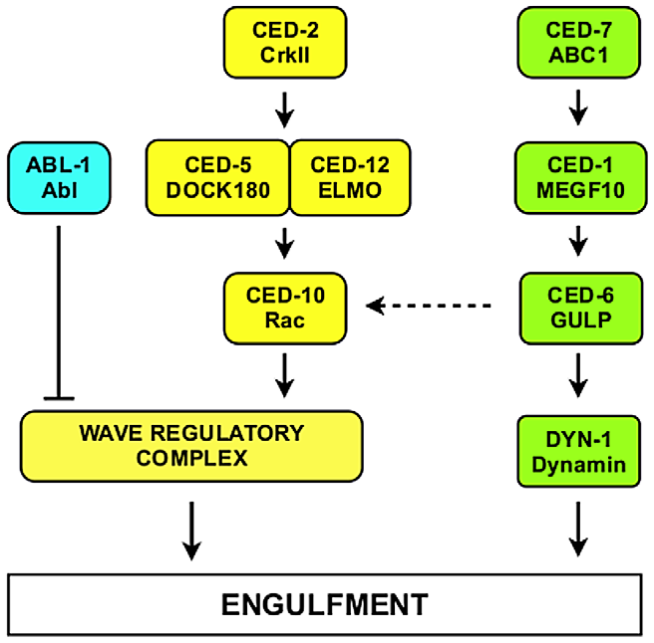
Core molecular pathways required for the engulfment of apoptotic cells. Proteins of the CED-10 Rac pathway are labeled in yellow. Proteins of the CED-1 pathway are labeled in green. *C. elegans* protein names are indicated above and their mammalian homologs below. The Wave Regulatory Complex (WRC) contains ABI-1, GEX-2, GEX-3, WVE-1 and NUO-3. The dashed arrow from CED-6 to CED-10 indicates that CED-1, CED-6 and CED-7 might also signal through CED-10 [6]. The CED-1 pathway is required for engulfment only, whereas the CED-10 Rac pathway is required for both engulfment and DTC migration.

In the pathway for membrane recruitment, which we refer to as the CED-1 pathway, four proteins have been identified, CED-7, CED-1, CED-6 and DYN-1 (Figure 1) [1]. These proteins activate DYN-1, a *C. elegans* dynamin homolog [2], which might recruit membrane for engulfment; in mammalian cells dynamin promotes extension of lamellipodial membrane protrusions [3].

The pathway for cytoskeletal rearrangement requires the small GTPase CED-10 Rac, the adapter protein CED-2 and the heterodimeric guanine nucleotide exchange factor CED-5/CED-12. CED-2 is thought to activate CED-5/CED-12, which, in turn, activates CED-10 Rac. Rac proteins are members of the Rho family of small GTPases that regulate the cytoskeleton and function in intracellular signaling [4]. CED-10 Rac activation causes actin cytoskeletal rearrangement and promotes engulfment [5], [6].

In addition to the two core engulfment pathways, more recent studies have identified a number of factors that regulate engulfment through these pathways. In *C. elegans*, MIG-2, the mammalian homolog of RhoG, another Rho family GTPase activates CED-5/CED-12 in parallel to CED-2 [7], [8]. The phosphatidylserine receptor PSR-1, the integrins INA-1 and PAT-3 and a WNT signaling pathway all appear to act upstream of CED-2 [9], [10]. In *Drosophila*, the Src protein Src42 and the non-receptor tyrosine kinase Shark act through the CED-1 Draper pathway [11]. Furthermore, Calcium release from the endoplasmic reticulum by a junctophilin-containing complex is also required for CED-1 Draper activity [12], [13].

Recently, we reported that the cytoskeletal regulatory protein ABI-1 is also an engulfment protein [14]. The mammalian homolog of ABI-1, Abi2, is found in a number of protein complexes, all of which regulate the actin cytoskeleton. One particular complex, the Wave Regulatory Complex (WRC) causes the formation of actin structures in response to activation by Rac [15], [16]. The WRC is composed of five proteins in *C. elegans*: WVE-1, GEX-2, GEX-3, ABI-1 and NUO-3. Soto et al. (2002) [17] and Patel et al. (2008) [18] presented evidence that suggested that GEX-2 and WVE-1, respectively, promote engulfment. Our genetic analysis, however, demonstrated that the CED-10 Rac pathway and ABI-1 act at least partially independently of each other. Our current model, based on all of these data is that the CED-10 Rac pathway activates the WRC but that there are other as yet unidentified molecular pathways that activate the WRC in parallel.

Far less studied are proteins that inhibit these two pathways. We showed that the tyrosine kinase and cytoskeletal regulator ABL-1 inhibits engulfment through ABI-1 in parallel to the CED-10 Rac pathway [14]. A small number of other proteins have been shown to inhibit apoptotic cell engulfment (compared to 25 proteins that promote engulfment). In mammalian cell culture, the small GTPase RhoA and its effector Rho-kinase have been shown to inhibit engulfment of apoptotic cells [19], consistent with the fact that RhoA and Rac oppose each other in many cellular processes. How Rho-kinase inhibits engulfment has not been demonstrated. In *C. elegans*, the Rac GTPase activating protein SRGP-1 inhibits engulfment by inactivating CED-10 [20]. The myotubularin lipid phosphatase MTM-1 and a CED-10 binding protein, SWAN-1, have also been shown to inhibit engulfment in *C. elegans* [21]–[23]. They are both proposed to act through the CED-10 Rac pathway. Recently, PGRN-1, a *C. elegans* progranulin has been shown to act in engulfment [24]. Notably, it is unclear how any of these proteins are regulated for their engulfment-inhibitory functions.

Cbl family proteins are E3 ubiquitin ligase and adaptor proteins with multiple cellular functions [25]. Cbl proteins consist of an N-terminal tyrosine kinase binding (TKB) domain followed by a conserved linker, then a RING finger domain and a C-terminal proline rich domain. The TKB domain is comprised of three subdomains: a 4-helix bundle, an EF hand and a modified SH2 domain. The crystal structure of the TKB domain has revealed that the three subdomains act together to bind to phosphotyrosines [26] and orient substrate proteins (usually tyrosine kinases) to allow the RING finger to promote their ubiquitination, targeting them for destruction or sequestration. Thus a major function of Cbl proteins is to downregulate signaling pathways in response to interactions with tyrosine phosphorylated signaling proteins [27]. Recent data show that Abi proteins are activated by epidermal growth factor (EGF) signaling and then in turn activate c-Cbl to polyubiquitinate the EGF receptor in a negative feedback regulatory loop [28]. In *C. elegans,* the Cbl homolog SLI-1 downregulates EGF signaling by causing ubiquitination of the LET-23 EGFR [29], [30], which decreases signaling from the downstream Ras homolog LET-60. Cbl has also been shown to interact with Rac, the CED-2-related protein Crk and Abl kinase [31]–[33]. We hypothesized that SLI-1 might act in engulfment pathways. In addition, we asked whether it did so by interacting with the *C. elegans* homologs of the above proteins.

We now present evidence that SLI-1 inhibits apoptotic cell engulfment. Surprisingly, we find that SLI-1 does so in parallel to the two core engulfment pathways and ABL-1 and independent of LET-60 Ras signaling. Lastly, we demonstrate that the ubiquitin ligase domain is partially dispensable for this process demonstrating that its tyrosine kinase-ubiquitinating function is unrelated to its mechanism of action in engulfment.

## Results

### SLI-1 inhibits apoptotic cell corpse engulfment

In animals with defects in apoptotic cell engulfment, the number of unengulfed corpses in the heads of first larval stage (L1) animals increases with the strength of the engulfment defect and defines a quantitative assay of engulfment defects [34]. L1 wild-type (N2) animals have no unengulfed corpses in their heads. Neither do animals with *sli-1* mutations alone. We used two alleles of *sli-1* in this study, *sy143* and *n3538* [35], [36]. *sy143* is a C to T transition that changes Gln152 to an amber stop codon; *n3538* is a C to T transition that changes Ser305 to Leu. To assess whether *sli-1* modulates apoptotic cell engulfment, we tested whether *sli-1* mutations suppressed or enhanced the engulfment defects of engulfment pathway genes.

The heads of animals containing a mutation in *sli-1* and null mutations in *ced-1* or *ced-7* or a strong mutation in *ced-6* (alleles *e1735*, *n1996* and *n2095*, respectively) had fewer unengulfed corpses than those with each of the engulfment mutants alone (Table 1). We did not test *dyn-1* mutants because they die during embryogenesis. Thus, SLI-1 appears to inhibit the engulfment of apoptotic cells. Alternative explanations for the effect of SLI-1 on these engulfment defects are presented in the next section of the paper. The fact that loss of *sli-1* function suppresses the engulfment defects caused by null *ced-1, ced-6* and *ced-7* mutation demonstrates that SLI-1 acts in parallel to or downstream of the CED-1 pathway.

**Table 1:** *sli-1* mutations suppress the engulfment defects caused by engulfment *ced* gene mutations.

Genotype	Corpses ± s.d.	*n*	*p-*Value
N2	0.05±0.23	19	
*sli-1(sy143)*	0.16±0.37	24	>0.2
*sli-1(n3538)*	0.04±0.2	25	>0.2
*ced-1(n2091)*	18.7±4.0	23	
*ced-1(n2091); sli-1(sy143)*	7.7±3.3	15	<0.0001
*ced-1(e1735)*	25.3±5.0	23	
*ced-1(e1735); sli-1(sy143)*	17.8±4.9	22	<0.0001
*ced-1(e1735); sli-1(n3538)*	17.5±3.8	21	<0.0001
*ced-2(n5101)*	15.8±4.9	25	
*ced-2(n5101); sli-1(sy143)*	18.5±6.9	26	<0.06
*ced-5(n1812)*	30.4±4.9	23	
*ced-5(n1812); sli-1(sy143)*	34.2±5.9	25	<0.01
*ced-5(n1812); sli-1(n3538)*	30.6±4.9	18	>0.2
*ced-6(n2095)*	26.5±4.1	24	
*ced-6(n2095); sli-1(sy143)*	14.2±4.6	15	<0.0001
*ced-6(n2095); sli-1(n3538)*	10.2±3.3	18	<0.0001
*ced-7(n1996)*	32.1±4.0	20	
*ced-7(n1996); sli-1(sy143)*	23.5±6.6	26	<0.0001
*ced-10(n1993)*	20.0±3.9	24	
*ced-10(n1993); sli-1(sy143)*	14.1±6.5	27	<0.0001
*ced-10(n1993); sli-1(n3538)*	13.8±5.3	25	<0.0001
*ced-12(tp2)*	20.8±3.7	22	
*ced-12(tp2); sli-1(sy143)*	17.3±4.7	17	<0.008
*ced-12(tp2); sli-1(n3538)*	20.3±6.0	25	>0.2
*ced-12(n3261)*	25.2±5.2	20	
*ced-12(n3261); sli-1(sy143)*	26.9±5.0	21	>0.2

First larval stage (L1) worms were anaesthetized and viewed using DIC microscopy. The numbers of cell corpses in the heads were counted. s.d., standard deviation.

Loss of *sli-1* function did not suppress the engulfment defects of null mutations in the CED-10 Rac pathway. Specifically, the engulfment defects of *ced-2(n5101), ced-5(n1812)* and *ced-12(n3261)* null mutants were not significantly modified by the presence of *sli-1(sy143)* or *sli-1(n3538)* mutations (Table 1). *ced-10* null mutants die during embryogenesis but we tested the effect of *sli-1* mutations on a partial loss-of-function allele, *ced-10(n1993). ced-10(n1993)* was suppressed by *sli-1*(lf) (for *sy143*, a decrease from 20.0 to 14.1 unengulfed corpses, *p*<0.0001; for *n3538,* a decrease to 13.8, *p*<0.0001). Suppression of a *ced-10* partial loss-of-function defect by *sli-1* mutations is consistent with a general inhibition of engulfment by *sli-1* but suppression of a partial loss-of-function mutation cannot be used to order genes within genetic pathways. In summary, *sli-1*(lf) was not able to suppress the engulfment defects caused by complete loss-of-function CED-10 pathway mutants. Thus, the CED-10 Rac pathway is unlikely to act by inhibiting SLI-1; rather, SLI-1 acts either parallel to or upstream of the CED-10 Rac pathway.

### SLI-1 does not affect the cell-death process directly

*sli-1* mutation might decrease the number of unengulfed cell corpses in engulfment mutants in a number of ways other than by suppressing apoptotic cell engulfment. *sli-1* mutation could (1) decrease programmed cell death, resulting in fewer cell corpses as is seen in *ced-3* caspase mutants [37], (2) alter the timing of corpse appearance during development like the protein CED-8 [38], resulting in fewer corpses at the time of observation, (3) alter cell-corpse morphology so that they could not be identified by DIC microscopy as corpses or, (4) cause the corpses to be unstable and lost rapidly.

To address whether *sli-1* normally prevents programmed cell death, we determined whether cells that are known to die by apoptosis normally during development do so in *sli-1* mutants. 16 cells undergo programmed cell death in the anterior pharynx during embryogenesis in wild-type animals [39]. The nuclei of these cells are identified easily using DIC microscopy [34]. Mutations in genes that normally cause cell death, such as *ced-3* or *ced-4*, have up to 14 extra recognizable cell nuclei in the anterior pharynx [34], [40]. *sli-1(sy143)* animals had no more nuclei than wild-type animals in their anterior pharynges (Table 2, *sli-1* mutation does not block cell death in the pharynx). To test for apoptosis defects more stringently, we observed whether *sli-1* mutation enhanced the death defect of a partial loss-of-function *ced-3* mutant *(n2427)* [41]. We observed no difference between *ced-3(n2427)* and *ced-3(n2427); sli-1(sy143)* animals (1.6 vs. 1.1 extra cells, Table 2, *sli-1* mutation does not block cell death in the pharynx).

**Table 2:** *sli-1* mutation and programmed cell death.

*sli-1* mutation does not block cell death in the pharynx			
Genotype	Extra cells ± s.d.	*n*	*p*-Value
N2	0.1±0.3	13	>0.2
*sli-1(sy143)*	0.1±0.3	13	
*ced-3(n2427)* a	1.6±1.4	13	>0.2
*ced-3(n2427); sli-1(sy143)* a	1.1±0.8	13	

*sli-1* suppresses the cell-killing effect of an engulfment gene			
Genotype	Extra cells ± s.d.	*n*	*p*-Value
*ced-12(tp2); ced-3(n2427)* a	5.4±1.3	10	<0.001
*ced-12(tp2); ced-3(n2427); sli-1(sy143)* a	2.8±1.5	10	

Third larval stage (L3) worms were anaesthetized and viewed using DIC microscopy. The numbers of extra cell nuclei in the anterior pharynges were counted.

a These strains contained *nIs96* [*lin-11p::gfp*] V.

**Figure 2:**
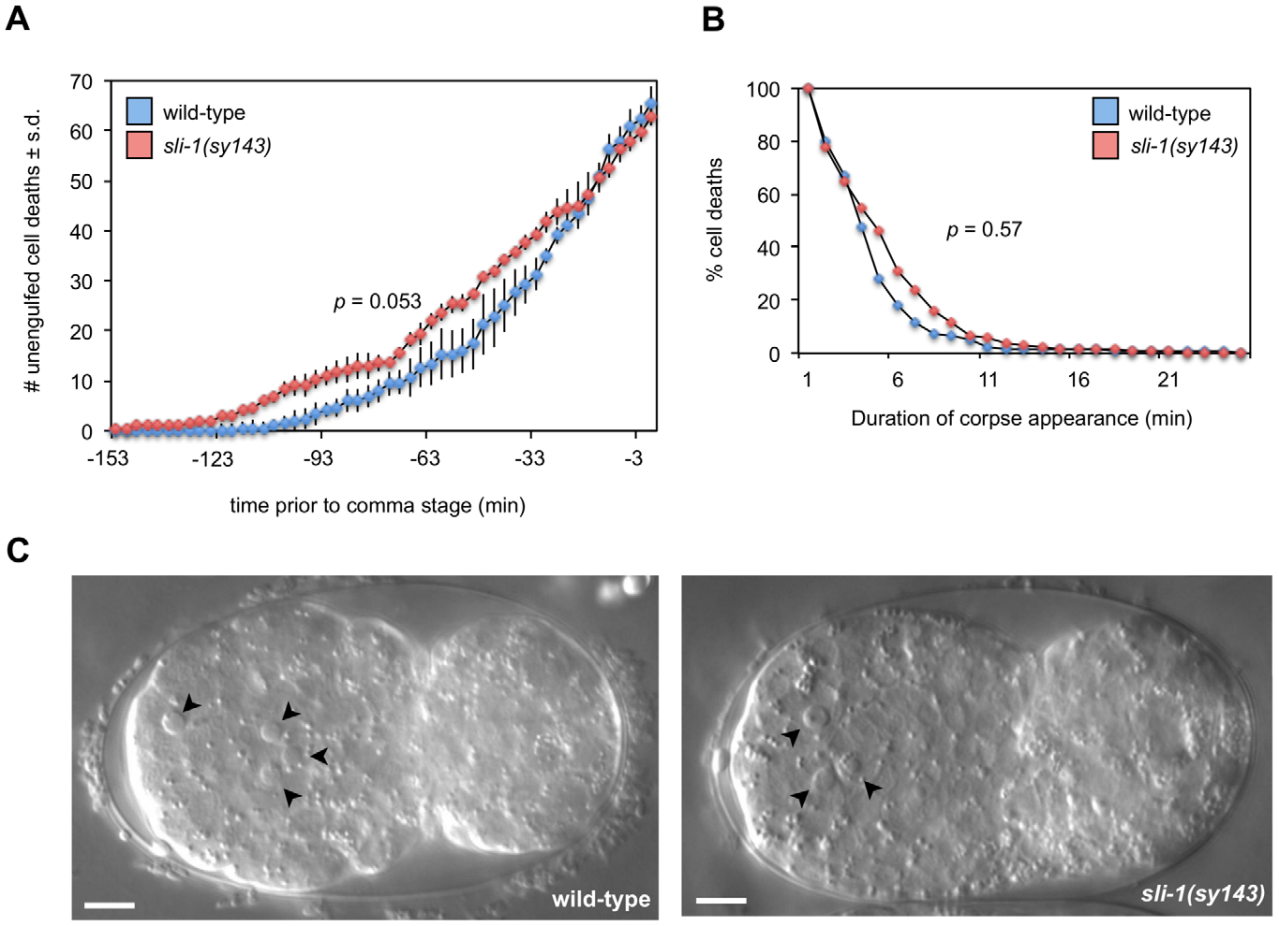
*sli-1* does not affect the timing of deaths or morphology of cell corpses. (A) The number and time of appearance of apoptotic cell corpses that occurred from the first cell death to the comma stage were recorded at 3-min intervals in wild-type and *sli-1(sy143)* animals using time-lapse DIC microscopy (see Materials and Methods). Mean numbers of corpses at each time point were calculated from three embryos for both wild-type and *sli-1(sy143)* animals. Statistical analysis was by the Wilcoxon rank-sum test. The curves are similar (*p*>0.05). (B) The duration of cell-corpse visibility is similar in wild-type and *sli-1(sy143)* embryos. The percentage of cell corpses visible for a given period was recorded. The duration of appearance of all cell corpses recorded from three wild-type (n = 159 cell corpses) and three *sli-1(sy143)* (n = 154) embryos was analyzed. Statistical analysis was by the Wilcoxon rank-sum test. The curves are similar (p>0.5). (C) The morphology of cell corpses in wild-type and *sli-1(sy143)* embryos are similar. Arrowheads, apoptotic corpses. Embryos were at similar stages of development, approximately six minutes prior to the comma stage. Bar = 5 microns.

We used time-lapse DIC microscopy to assess whether *sli-1* loss-of-function affected the timing, persistence or morphology of cell corpses. The development of wild-type and *sli-1(sy143)* animals was recorded for approximately 150 minutes. We found that *sli-1(sy143)* animals developed on average more slowly than wild-type animals. To account for the difference in the rate of development, we counted the number of cell deaths that occurred from the first cell death up to the comma stage. *sli-1(sy143)* worms take approximately 31 minutes longer than wild-type animals to develop to that stage at 20°C (144 minutes compared to 103 minutes). During this time, approximately 60–65 cell corpses appear in the wild-type animal. The number of cell corpses that appeared and when they appeared in wild-type and *sli-1(sy143)* embryos did not differ significantly (Figure 2A). However, the timing of appearance approaches statistical significance (*p* value = 0.053), probably related to the difference in developmental speed. The length of time that corpses persisted was similar in wild-type and *sli-1(sy143)* animals (Figure 2B). In addition, apoptotic cell corpses in wild-type and *sli-1(sy143)* animals looked similar (Figure 2C). We conclude that the morphology and time of appearance of apoptotic cell corpses is not affected by *sli-1* mutation.

### *sli-1* is broadly expressed during embryogenesis

To study the expression pattern of SLI-1, we expressed *gfp* under control of the *sli-1* promoter. Specifically, we fused the 5000 bp 5′ of the *sli-1* ATG to *gfp* and injected that construct into wild type (N2) worms. Fluorescence was seen broadly throughout the embryo beginning prior to gastrulation and continuing through the L1 stage (Figure S1). Higher levels of expression were seen in cells that would form the head beginning at approximately the 1½ fold stage of embryonic development. This pattern continued through the first larval stage with L1 animals showing GFP expression at high levels in the head and at lower levels throughout the body, including in body wall muscles, hypodermis, intestine, anal depressor muscles, and several neurons. During later larval development expression is seen in the distal tip cells (DTCs) (Figure S1 panel ix). In adults, GFP was found in the head, body wall muscles, hypodermis, DTCs and some neurons. This expression pattern is consistent with our results; we observe *sli-1*-dependent phenotypes in the heads and DTCs (see next section in results).

**Figure 3:**
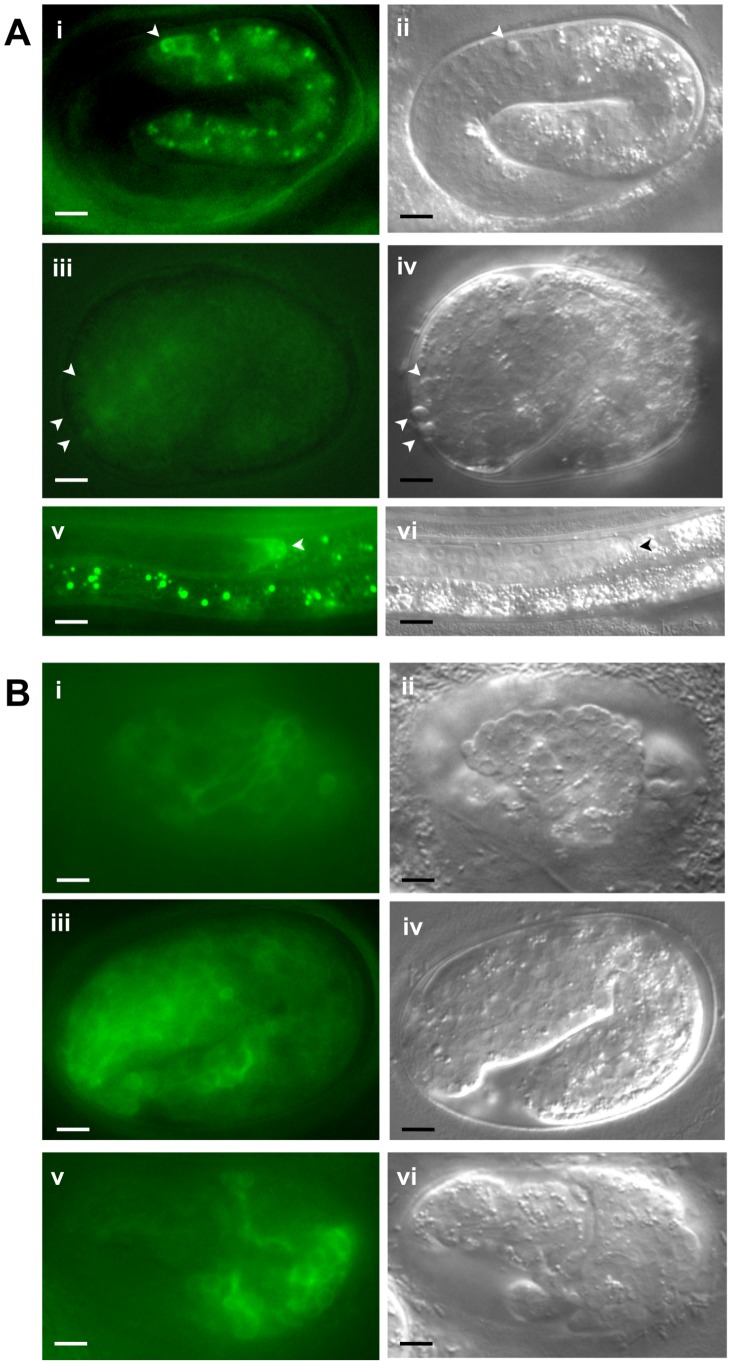
Expression pattern of SLI-1::GFP under control of the *sli-1* promoter. SLI-1::GFP was expressed under the control of its own promoter in an extrachromosomal array. (A) Images of morphologically normal animals expressing the SLI-1::GFP transgene. i, iii and v show fluorescence images and ii, iv and vi show accompanying DIC images (i–iv, embryos; v–vi, L4 larvae). Arrowheads in i–iv indicate positions of unengulfed cell corpses. In image iii corpses are not visible. Arrowheads in v and vi indicate the DTC. (B) Images of morphologically abnormal embryos expressing the SLI-1::GFP transgene. i, iii and v show fluorescence images and ii, iv and vi show accompanying DIC images. Arrowheads indicate positions of unengulfed cell corpses. Bar = 5 microns.

We also generated a translational fusion with *sli-1* containing a C-terminal *gfp* expressed under control of the *sli-1* promoter. This transgene was injected as an extrachromosomal array into *ced-10(n1993); sli-1(sy143)* animals. In animals in which high levels of GFP were observed, the animals invariably died during embryogenesis with bizarre morphological defects, indicating that overexpression of *sli-1* is toxic to worms (Figure 3B). However, in animals with low levels of SLI-1::GFP expression, morphological abnormalities were not seen. We found that in these low level SLI-1::GFP expressing animals, the *sli-1* mutant engulfment phenotype was rescued (data not shown).

We analyzed the expression pattern of P*_sli-1_sli-1::gfp* in animals that were morphologically normal. The expression profile of this transgene was quite similar to that of the transcriptional fusion but the expression level was far lower (Figure 3A). Interestingly, SLI-1::GFP was observed surrounding cell corpses in most transgenic animals though in a small minority of cell corpses in each animal (Figure 3A, panel i). While this finding suggests that SLI-1 normally is found at the cell-cell interface at some point during the engulfment process, there are several caveats, some of which argue against and others for this interpretation. SLI-1 might not normally surround cell corpses and only does so in animals in which the SLI-1::GFP transgene is overexpressed (although we suspect that it is not overexpressed at that high a level in morphologically normal animals, as we noted above). Another fact that appears inconsistent with SLI-1 normally being present at the interface between the engulfing and engulfed cell is that most corpses seen on DIC were not surrounded by GFP haloes. However, at least two phenomena could account for the lack of more GFP haloes. First, the embryos where we could analyze unengulfed cells had comparatively low levels of SLI-1::GFP expression, which would decrease the sensitivity of the assay. Second, since SLI-1 inhibits engulfment, it might need to be removed from the cell-cell interface for engulfment to occur. Thus, SLI-1 might only surround cell corpses briefly before being relocated within the engulfing cell.

In mammals, the SLI-1 homolog Cbl is found primarily in the cytoplasm, but also at the plasma membrane and bound to the cytoskeleton [25]. In our transgenic lines in which SLI-1::GFP was overexpressed at high levels, GFP was seen preferentially at the cell periphery and less so in the cytoplasm (Figure 3B) though it is unclear if this localization is physiological given the overexpression. Furthermore, these embryos were very abnormal morphologically so conclusions regarding subcellular localization in these animals should be made very cautiously.

Because high levels of SLI-1::GFP cause embryonic lethality we were unable to test whether unengulfed cell corpses accumulated in animals with high levels of SLI-1::GFP. However we did analyze animals with low levels of SLI-1::GFP. We observed the number of unengulfed cell corpses in N2 embryos that were morphologically normal at the two-fold stage and contained the *sli-1::gfp* transgene and compared them to N2 animals without the transgene. Embryos that contained the transgene had 9.2 unengulfed corpses vs. 8.1 for animals without the transgene (Table S1). While this difference is statistically significant (*p*<0.04), it is unclear if this represents a biologically significant difference; this is not surprising since the amount of overexpression appears to be low. At hatching there was no significant difference in the number of unengulfed cell corpses between animals with or without the transgene.

### *sli-1* mutation suppresses other defects associated with engulfment pathway genes

Studies of *C. elegans* mutants partially defective in programmed cell death (such as partial loss-of-function *ced-3* mutants) demonstrated that engulfment dysfunction can enhance apoptotic defects [41], [42]. These studies concluded that engulfment of dying cells promotes their apoptosis. Similar promotion of cell death by engulfment has been observed in *Drosophila* [43], indicating that the cell-killing effect of engulfment is evolutionarily conserved.

In partial loss-of-function *ced-3* mutants, such as *n2427*, some of the cells fated to die will begin the dying process (based on morphological appearance) but then recover and survive. However, in animals with engulfment gene mutations as well as partial *ced-3* loss-of-function mutations a much larger percentage of cells normally fated to die survive.

We compared the number of extra nuclei in the pharynges of *ced-12(tp2); ced-3(n2427)* and *ced-12(tp2); ced-3(n2427); sli-1(sy143)* animals to determine if *sli-1* loss-of-function could suppress the apoptotic defect of an engulfment pathway mutation. Fewer extra nuclei were seen in animals that contained the *sli-1(sy143)* mutation (Table 2, *sli-1* suppresses the cell-killing effect of an engulfment gene), demonstrating that SLI-1 suppresses the cell-killing promoted by engulfment genes, consistent with it engulfment suppression role.

**Figure 4:**
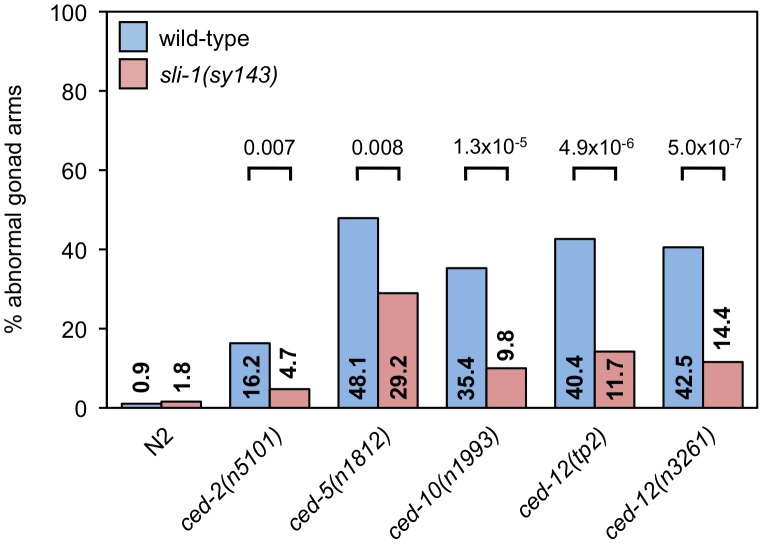
*sli-1* mutation suppresses the DTC migration defects caused by all CED-10 Rac pathway gene mutations. The gonads of young adult animals mutant for an engulfment gene with or without *sli-1* mutation were observed and scored for morphology using DIC microscopy. Scoring was as described in Materials and Methods. Percentages of abnormal gonad arms are shown. More than 80 gonad arms were scored for all genotypes. *p* values derived using Fisher's exact test are shown.

The two distal tip cells (DTCs) migrate during development from the center of the animal outward and then back again, meeting approximately in the center of the animal. As they move, the gonads form behind them, resulting in two U-shaped gonads [44], [45]. In *ced-10* Rac pathway mutants, the gonads often have extra turns or arms caused by abnormal DTC migration [46]. We tested whether *sli-1* mutation suppressed the DTC migration defects of *ced-10* Rac pathway mutants. Mutation of *sli-1* decreased the percentage of gonadal morphology defects in all *ced-10* Rac pathway mutants tested, including null *ced-5* and *ced-12* mutants (Figure 4). 48% of the gonads of *ced-5(n1812)* animals were abnormal whereas only 29% of the gonads of *ced-5(n1812); sli-1(sy143)* animals were abnormal (*p*<0.008), while the percentages of abnormal gonads in *ced-12(n3261)* and *ced-12(n3261); sli-1(sy143)* animals were 40% and 12%(*p*<4.9×10^−6^). These data demonstrate that SLI-1 inhibits DTC migration and that it does so independent of the CED-10 Rac pathway. Notably, since the CED-1 pathway has no role in DTC migration, SLI-1 appears to act in parallel to both engulfment pathways.

In summary, loss of *sli-1* suppresses *ced-10* Rac pathway DTC migration defects but does not suppress *ced-10* Rac pathway engulfment defects. At least two models could account for these findings. SLI-1 could act through one molecular pathway to inhibit apoptotic cell engulfment (e.g. the CED-10 Rac pathway) and through another molecular pathway to inhibit DTC migration. Alternatively, SLI-1 might act in a common pathway to inhibit both engulfment and migration but the relative importance of that pathway might be much greater in DTC migration than in engulfment. This difference would account for the ability of *sli-1* mutation to suppress CED-10 Rac pathway DTC migration defects but not CED-10 Rac pathway engulfment defects.

For example, the CED-10 Rac pathway and another SLI-1-inhibited pathway might both promote DTC migration and either pathway alone is sufficient for normal DTC migration. If this were the case, loss of SLI-1 function would derepress the SLI-1-regulated pathway and suppress DTC migration defects even if the CED-10 Rac pathway were completely non-functional, as we observed. Engulfment, however, might be totally dependent on the CED-10 Rac pathway. In this case, even if *sli-1* loss-of-function derepressed the other parallel pathway, the defect caused by loss of the CED-10 Rac pathway might not be able to be overcome by derepression of the SLI-1-regulated pathway. We favor this model (i.e. that SLI-1 acts on the same parallel pathway in both engulfment and DTC migration) both because of its parsimony and because of data we will present later in the paper (See last paragraph of the section titled SLI-1 acts independently of ABL-1).

### SLI-1 probably acts in engulfing cells

To determine whether SLI-1 function is required in the dying cell or the engulfing cell, we used *sli-1* mutant animals containing a *sli-1* transgene that was expressed under the control of heat shock promoters (protocol adapted from Wu and Horvitz (1998) [46]). Specifically, the number of cell corpses in the heads of newly hatched worms was counted within 300 minutes of heat shock. Since all apoptotic deaths in the heads occur prior to 300 minutes before hatching, *sli-1* could not be expressed in the dying cells.

Expression of *sli-1* in *ced-10(n1993); sli-1(sy143)* animals increased the number of unengulfed corpses in L1 heads from 13.4 to 23.7 (*p*<1×10^−4^) (for comparison, *ced-10(n1993)* animals had 20.0 corpses (Table 1)), whereas expression of a *gfp*-only control transgene did not increase the number of unengulfed corpses (17.3 vs. 16.4; *p*>0.2) (Table 3). Notably, in the *gfp*-expressing animals, GFP was not seen in the cell corpses, in support of our hypothesis that the engulfed cell did not make new proteins (data not shown). Thus, expressing *sli-1* outside of the engulfed cell rescues the *sli-1* mutant phenotype, indicating that *sli-1* acts in the engulfing cell.

**Table 3:** Overexpression of *sli-1* reverses the effect of *sli-1(sy143)* on engulfment in *ced-10(n1993); sli-1(sy143)* animals.

Transgene	Heat Shock	Corpses ± s.d.	n	*p*-Value
P*_hsp_gfp*	−	17.3±6.1	25	>0.2
P*_hsp_gfp*	+	16.4±6.7	23	
P*_hsp_sli-1wt*	−	13.4±4.2	34	<1×10^−4^
P*_hsp_sli-1wt*	+	23.7±6.2	27	
P*_hsp_sli-1ΔRING*	−	13.9±4.7	20	<0.006
P*_hsp_sli-1ΔRING*	+	20.0±6.9	15	

*ced-10(n1993); sli-1(sy143)* embryos containing the transgenes indicated above were heat shocked at 33°C for 1 hour and then allowed to recover for 3–3.5 hours at 20°*C.* The numbers of cell corpses in the heads of anaesthetized first larval stage (L1) worms were counted using DIC microscopy within 0.5 h of hatching. The number of persistent corpses was determined from two independent transgenic lines for each transgene except for P*_hsp_sli-1ΔRING* (see text for explanation). s.d., standard deviation.

### SLI-1 acts independently of ABL-1

Five proteins have been identified in *C. elegans* that inhibit the engulfment of apoptotic cells. Three of them, the myotubularin lipid phosphatase MTM-1, the adapter SWAN-1 and the RacGAP SRGP-1, act through the CED-10 Rac pathway [20]–[23]. It is unknown how the *C. elegans* progranulin, PGRN-1, suppresses engulfment defects [24]. Genetic and biochemical data indicate that ABL-1 inhibits ABI-1 in parallel to the CED-10 Rac pathway [14].

Since *abl-1* and *sli-1* both act independently of the *ced-10* Rac pathway, we asked whether *sli-1* and *abl-1* act in the same pathway. We generated triple mutant strains containing mutations in an engulfment gene and in *abl-1* and *sli-1* and compared the engulfment defects and DTC migration defects to those of double mutant strains containing mutations in engulfment genes and either *abl-1* or *sli-1*. We found that the engulfment defect of the null mutant *ced-1(e1735)* was suppressed to a greater degree by the combination of *abl-1(ok171)* and *sli-1(n3538)* than by either mutation alone (Table 4). The same phenomenon was observed for the partial loss of function *ced-10(n1993)* allele. The engulfment defect of the null mutant *ced-5(n1812)* was not suppressed by the *abl-1* or *sli-1* mutations together or alone, consistent with our prior results that neither *sli-1* nor *abl-1* loss-of-function can suppress null defects in the *ced-10* Rac pathway. The *ced-6(n2095)* engulfment defect was suppressed by both the *abl-1(ok171)* and the *sli-1(n3538)* alleles, but they did not enhance each other. The *ced-6(n2095); sli-1(n3538)* strain had 10.2 unengulfed corpses while the *ced-6(n2095); abl-1(ok171) sli-1(n3538)* strain had 10.4 unengulfed corpses. While it is not clear why these mutations did not enhance each other in the *ced-6* mutant background, the suppression by *sli-1(n3538)* is very strong and we suspect that we are near the threshold of the sensitivity of the engulfment assay so that further enhancement cannot be detected despite independent effects on engulfment.

**Table 4:** Losses of *sli-1* and *abl-1* enhance each other's suppression of some engulfment gene defects.

Genotype	Corpses ± s.d.	*n*	*p-*Value
*ced-1(e1735)*	25.3±5.0	23	
*ced-1(e1735); sli-1(n3538)*	17.5±3.8	21	0.0049
*ced-1(e1735); abl-1(ok171)*	21.3±5.0	20	<0.0001
*ced-1(e1735); abl-1(ok171) sli-1(n3538)*	14.2±4.4	24	
*ced-5(n1812)*	30.4±4.9	23	
*ced-5(n1812); sli-1(n3538)*	30.6±4.9	18	>0.2
*ced-5(n1812); abl-1(ok171)*	28.4±4.4	20	>0.2
*ced-5(n1812); abl-1(ok171) sli-1(n3538)*	29.7±4.8	22	
*ced-6(n2095)*	26.5±4.1	24	
*ced-6(n2095); sli-1(n3538)*	10.2±3.3	18	>0.2
*ced-6(n2095); abl-1(ok171)*	20.2±3.5	20	<0.0001
*ced-6(n2095); abl-1(ok171) sli-1(n3538)*	10.4±2.3	21	
*ced-10(n1993)*	20.0±3.9	24	
*ced-10(n1993); sli-1(n3538)*	13.8±5.3	25	0.0011
*ced-10(n1993); abl-1(ok171)*	12.1±4.4	20	0.016
*ced-10(n1993); abl-1(ok171) sli-1(n3538)*	8.9±4.6	19	

First larval stage (L1) worms were anaesthetized and viewed using DIC microscopy. The numbers of cell corpses in the heads were counted. s.d., standard deviation. *p-*Values refer to comparisons between the double mutant and accompanying triple mutant strains.

**Figure 5:**
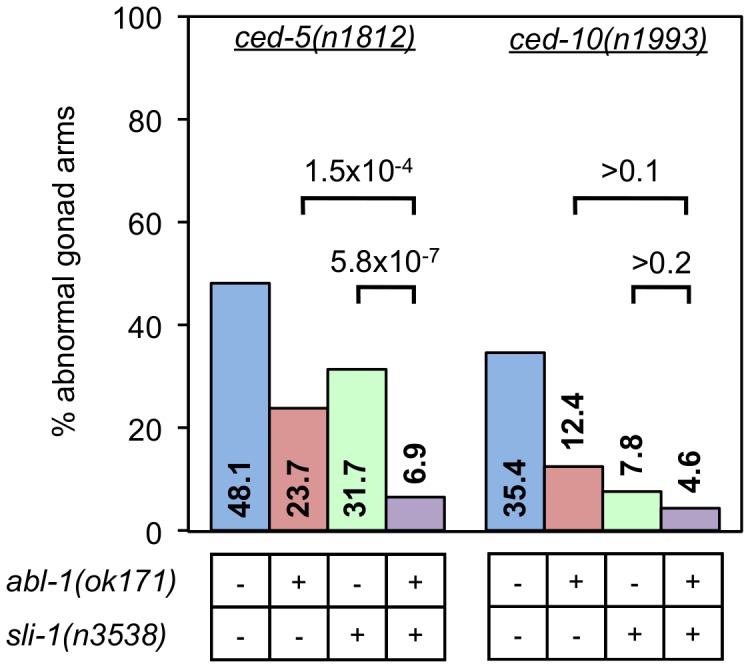
Losses of *sli-1* and *abl-1* enhance each other's suppression of some engulfment gene DTC defects. The gonads of *ced-5(n1812)* and *ced-10(n1993)* young adult worms with and without mutations in *abl-1(ok171)* and/or *sli-1(n3538)* were scored for morphology using DIC microscopy. More than 80 gonad arms were scored for all genotypes. *p* values derived using Fisher's exact test are shown.

For the DTC migration defect, *ced-5(n1812)* was suppressed by both *abl-1(ok171)* and *sli-1(n3538)* and was significantly more suppressed by the combination of the two mutations (Figure 5). By contrast, the DTC migration defect of *ced-10(n1993)* was suppressed so effectively by *sli-1(n3538)* that the addition of the *abl-1(ok171)* mutation did not enhance the suppression, similar to what was observed in engulfment with the *ced-6(n2095)-*containing strains. However, it appears that there is a trend towards increased suppression with *sli-1* and *abl-1* mutations together though the difference does not reach statistical significance (Figure 5).

**Figure 6:**
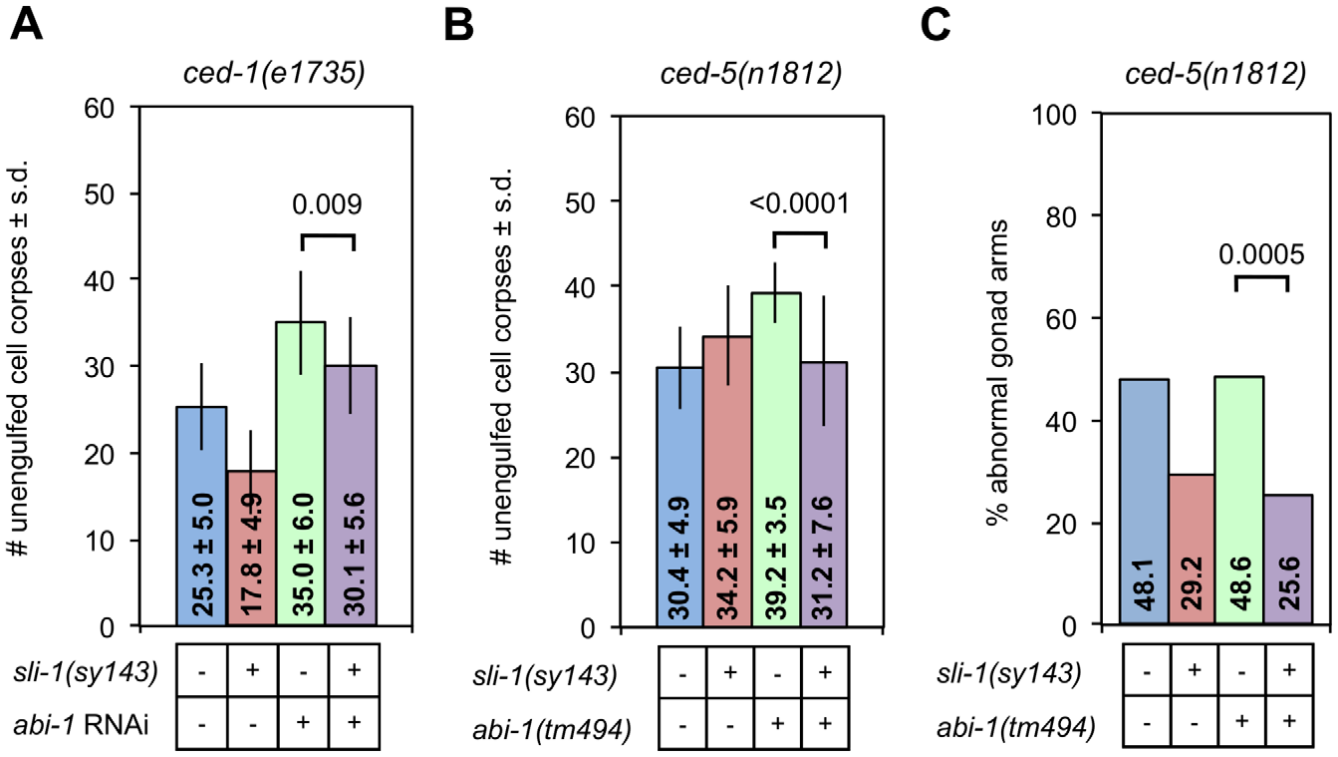
Loss of *sli-1* function suppresses engulfment and DTC migration defects in an *abi-1* loss-of-function mutant. (A) The heads of *ced-1(e1735)* L1 animals with or without the *sli-1(sy143)* mutation and with or without treatment with *abi-1* feeding RNAi were scored for engulfment defects using DIC microscopy. The mean ± standard deviation (s.d.) is indicated within each bar. Error bars are sd. *p* values derived using Student's t test are shown. At least 15 animals were analyzed for each genotype. (B) The heads of *ced-5(n1812)* L1 animals with or without the *sli-1(sy143)* and/or *abi-1(tm494)* mutations were scored as in (A). At least 15 animals were analyzed for each genotype. (C) The gonads of *ced-5(n1812)* young adult worms with and without mutations in *sli-1(sy143)* and/or *abi-1(tm494)* were scored for morphology using DIC microscopy. More than 80 gonad arms were scored for all genotypes. *p* values derived using Fisher's exact test are shown.

*abi-1* encodes the only *C. elegans* homolog of Abi, a member of the Wave Regulatory Complex (WRC). A combination of genetic and biochemical data suggest that ABL-1 and the CED-10 Rac pathway both act on the WRC through ABI-1 in parallel to each other: CED-10 Rac activates ABI-1 and ABL-1 inhibits it. Since SLI-1 acts in parallel to ABL-1, we asked whether it also acts on ABI-1. The only *abi-1* mutations in existence (and *abi-1* feeding RNAi) are quite weak and have no effect on engulfment alone but do enhance the engulfment defects of mutations in other engulfment genes. Therefore, we analyzed the effects of *abi-1* mutation in combination with another engulfment mutation. Specifically, *ced-1(e1735)* null mutant animals containing combinations of mutations of *abi-1* and/or *sli-1* were assessed for the magnitude of their engulfment defects. *sli-1(sy143)* suppressed the engulfment defect of *ced-1(e1735)* animals in the presence or absence of the *abi-1(tm494)* mutation (Figure 6A). *ced-1(e1735)* L1 animals had 25.3 unengulfed corpses and *ced-1(e1735); abi-1(tm494)* animals had 35.0 corpses. *ced-1(e1735); abi-1(tm494); sli-1(sy143)* animals had 30.1 corpses. Similar findings were found for *ced-5(n1812)* mutants (Figure 6B). We also tested the effect of *abi-1* on DTC migration using the *ced-5(n1812)* null mutation (Figure 6C). Similar to the findings with *ced-1* in engulfment, *sli-1(sy143)* suppressed the DTC migration defect of *ced-5(n1812)* (48% vs. 29%) and *sli-1(sy143)* suppressed the DTC migration defect of an *abi-1(tm494); ced-5(n1812)* double mutant (49% vs. 26%). Thus, mutation of *abi-1* did not completely suppress the effect of *sli-1* on engulfment or DTC migration. *abi-1(tm494)* abolishes the ability of *abl-1* null mutations to suppress defects in engulfment and DTC migration [14]. While these results do not prove that *sli-1* acts in a different pathway from *abi-1*, the findings are in stark contrast to those for *abl-1,* since *abi-1* mutation does not abrogate the effects of a *sli-1* null mutation on engulfment and DTC migration. Thus, *abi-1* might act independently of the WRC.

The finding that *sli-1(sy143)* suppresses the *abi-1(tm494)* engulfment defect in the presence of a *ced-5(n1812)* null mutation (Figure 6B) supports our model that *sli-1* acts in parallel to the *ced-10* Rac pathway rather than upstream of the *ced-10* Rac pathway in engulfment. The *ced-5(n1812)* mutation totally inactivates the *ced-10* Rac pathway. If *sli-1* acted upstream of the *ced-10* Rac pathway, the *ced-5(n1812)* mutation would block the ability of *sli-1(sy143)* to suppress the *abi-1(tm494)* engulfment defect, which we did not observe.

### SLI-1 acts independently of LET-60 Ras

**Figure 7:**
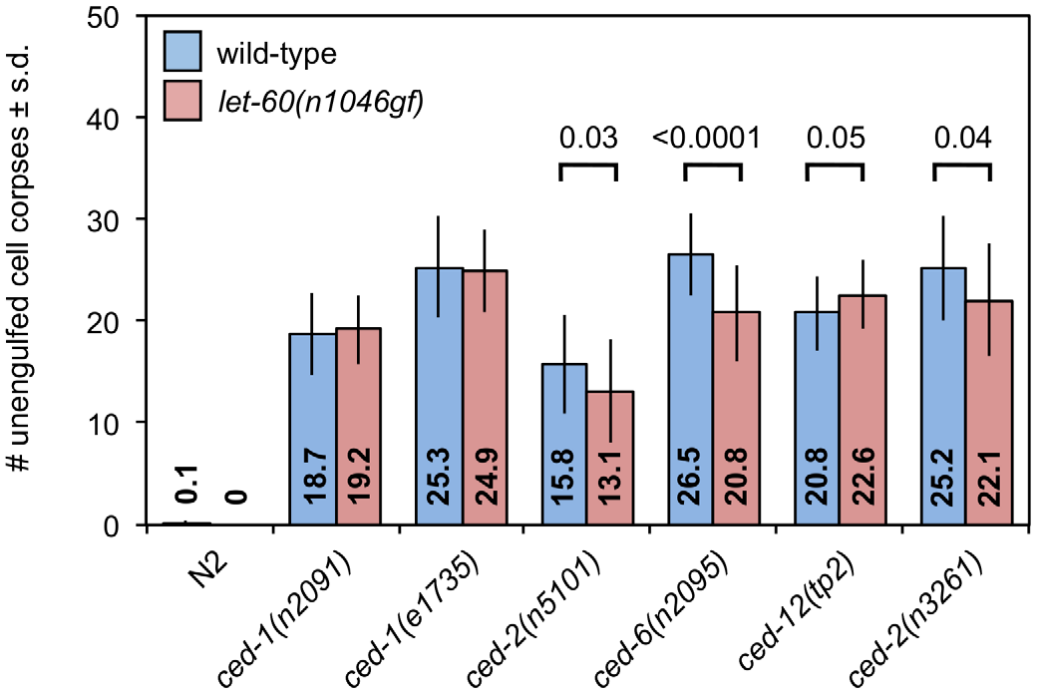
A *let-60* Ras gain-of-function mutation does not affect apoptotic cell engulfment.

SLI-1 inhibits the LET-23 EGFR/LET-60 Ras pathway and is thought to do so by ubiquinating the LET-23 protein, targeting it either for destruction or sequestration [29], [30]. Mammalian Ras activates Rac. Therefore, it was plausible that SLI-1 might inhibit engulfment by suppressing the LET-23/LET-60 pathway and consequently decreasing activation of CED-10 Rac by LET-60. To test this possibility, we generated strains doubly mutant for engulfment genes and the gain-of-function mutation *let-60(n1046gf)*. We would expect gain-of-function mutations in this pathway to suppress engulfment defects if *sli-1* normally inhibits this pathway. We found no consistent effect on the number of unengulfed apoptotic cells in animals with or without the *let-60(n1046gf)* mutation (Figure 7). One allele of *ced-12* was slightly enhanced while another allele of *ced-12* and an allele of *ced-2* were slightly suppressed. The only significantly modulated mutation was *ced-6(n2095)*, which was suppressed. Possibly this effect reflects a gene- or allele-specific interaction with *let-60.* Regardless, this pattern does not phenocopy either *sli-1* mutation. Thus, *sli-1* does not appear to act through the *let-23* EGFR/*let-60* Ras pathway to inhibit engulfment.

### SLI-1 uses the TKB domain to affect engulfment and migration

**Figure 8:**
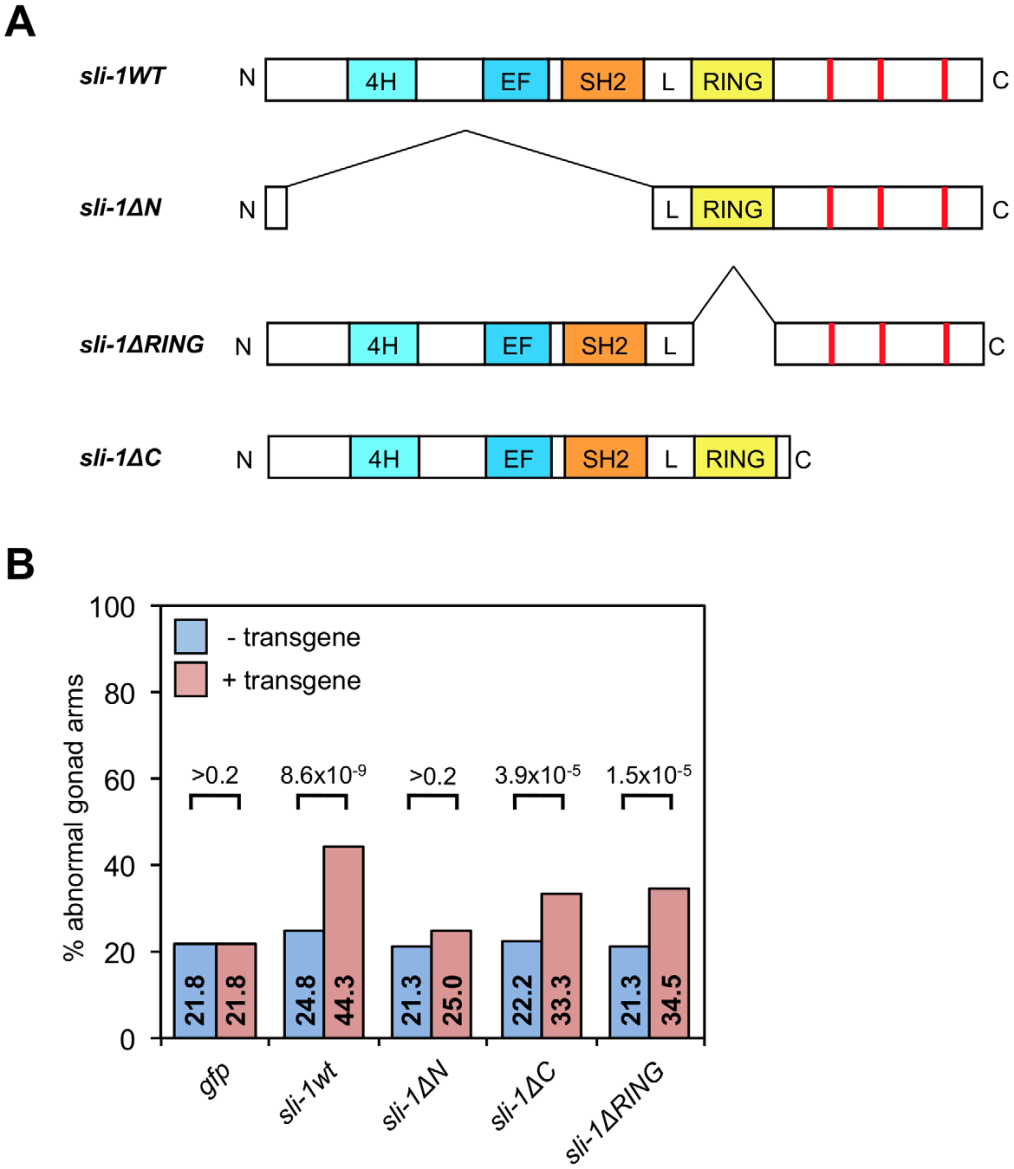
The TKB domain of *sli-1* is required for its cell migration function. (A) Truncation minigene *sli-1* constructs used in this study. Red lines represent proline-rich areas. L, conserved linker domain between the TKB domain (composed of the 4H, EF hand and SH2 domains) and the RING finger. (B) The gonads of *ced-10(n1993); sli-1(sy143)* young adult animals with or without transgenic arrays containing the genes shown in (A) were scored for abnormal morphology after heat shock. 200 gonad arms in two independent strains were analyzed per genotype. *p* values derived using Fisher's exact test are shown.

To determine which domain of SLI-1 is required for its suppression of engulfment and DTC migration defects, we ectopically expressed truncated forms of SLI-1 under control of the *C. elegans* heat-shock promoters in *sli-1* mutant animals. The SLI-1 protein contains three domains, an N-terminal domain that binds tyrosine kinases (and several other proteins), a RING finger, which mediates its E3 ubiquitin ligase function and a C-terminal domain, which contains several proline-rich regions. Minigenes encoding wild-type *sli-1* and truncation mutants of *sli-1* lacking each of the three domains expressed under heat-shock promoter control were injected into *ced-10(n1993); sli-1(sy143)* worms. These constructs were generated previously [30] and generously provided to us by Paul Sternberg. *ced-10(n1993); sli-1(sy143)* larvae harboring extrachromosomal arrays were incubated for one hour at 33°C, and their gonadal morphologies were analyzed 30 hours later in young adults. The arrays contained *sli-1* minigenes encoding full-length *sli-1* or *sli-1* lacking the N-terminus, RING finger or C-terminus (*sli-1wt, sli-1ΔN, sli-1ΔRING* or *sli-1ΔC,* respectively). Figure 8 shows that the *sli-1wt* construct rescued the defect completely, while *sli-1ΔRING* and *sli-1ΔC* both partially rescued the defect and the *sli-1ΔN* did not rescue the defect at all. We also tested the *sli-1ΔRING* transgene in engulfment and found that it partially rescued the engulfment suppression defect (Table 3).

Thus, the N-terminal tyrosine kinase binding domain was strictly required for the function of *sli-1* in DTC migration, whereas the RING finger and C-terminus were at least partially dispensable, suggesting that the ubiquitin ligase activity is unlikely to be central to the role of *sli-1* in DTC migration. Consistent with our findings in DTC migration, the RING finger was also partially dispensable in engulfment.

## Discussion

We have demonstrated that SLI-1 negatively regulates the engulfment of apoptotic cells. *sli-1* inhibits the engulfment process as well as the migration of distal tip cells during gonadogenesis and the engulfment-related cell-killing process. Our genetic analysis suggests that SLI-1 acts in a manner that does not require the known engulfment pathways. Ectopic expression experiments indicate that SLI-1 acts in engulfing cells and that its function is dependent on its N-terminal tyrosine kinase binding domain. Interestingly, these experiments demonstrate that the ubiquitin ligase function of SLI-1 is at least partially dispensable.

In mammals, the SLI-1 homolog Cbl interacts physically interacts with the CED-2-related protein Crk, Abl, Abi2 and regulates the activity of the CED-10 homolog Rac [28], [31]. In addition, in both mammals and worms, SLI-1 Cbl downregulates LET-23 EGFR by ubiquitination [29]. These interactions provided the rationale for our study of SLI-1 in engulfment initially. However, we found that the effects of SLI-1 on engulfment were independent of all of these proteins (with the possible exception of ABI-1; we were unable to test an *abi-1* null mutant). This finding highlights the multiple roles signaling proteins play in the regulation of complex cell biological processes. Also, these data emphasize the value of genetic analyses in discerning the physiological relevance of physical interactions discovered *in vitro* for a particular process.

Like many other genetic suppressors, *sli-1* mutation has no effect on normal engulfment. Specifically, only two engulfment suppressors, *srgp-1* and *pgrn-1,* have been shown to increase the rate of clearance of apoptotic cells in wild-type animals whereas *abl-1, swan-1* and *mtm-1* do not do so [14], [20]–[24]. Notably, the *srgp-1* and *pgrn-1* effects are subtle ones seen in early embryos. Possibly, the engulfment process is so efficient that derepressing it by removing inhibitors has little or no demonstrable effect. Similarly, only *srgp-1* and *mtm-1* cause engulfment defects when overexpressed. However, overexpression of a protein does not always result in increased activity; activation of the protein might be required, explaining the lack of overexpression phenotypes. In the case of *sli-1,* overexpression is toxic to worms so our ability to discern whether overexpression caused increased cell corpse accumulation was limited.

The discovery of SLI-1 as an inhibitor of engulfment adds to the small list of engulfment inhibitory proteins. Moreover, our genetic analysis puts SLI-1 into a new genetic pathway. Specifically, *sli-1* loss-of-function mutations suppress the engulfment defects of *ced-1* pathway null mutations and the DTC migration defects of *ced-10* Rac pathway null mutations. Thus, SLI-1 could act in a molecular pathway in parallel to both the *ced-1* and *ced-10* Rac pathways or it might act downstream of one or both pathways. However, the *ced-1* pathway has no role in DTC migration, so it is unlikely that *sli-1* acts downstream of the *ced-1* pathway given its effect on that process. Also, *sli-1* loss-of-function mutations do not suppress the engulfment defects of *ced-10* Rac pathway null mutations and therefore cannot be downstream of the *ced-10* Rac pathway. Thus, the simplest model consistent with the data is that *sli-1* acts in parallel to both *ced-10* Rac and *ced-1* pathways.

*abl-1,* another inhibitor of engulfment and DTC migration defects, has a very similar pattern of interactions with the two core engulfment pathways, demonstrating that it, too, acts in parallel to the *ced-1* and *ced-10* Rac pathways. We show that *abl-1* and *sli-1* act in parallel to each other in these processes as well. Thus, SLI-1 defines a new pathway of inhibition of engulfment and DTC migration.

The genetic interactions between *abl-1* and *abi-1* and *sli-1* and *abi-1* differ considerably. Whereas even very weak loss-of-function of *abi-1* completely suppresses the effects of *abl-1* mutations on engulfment and DTC migration, the same *abi-1* mutation only minimally suppresses the effect of *sli-1* on these processes. These findings are consistent with a model in which *sli-1* acts independently of the Wave Regulatory Complex in engulfment and DTC migration though we cannot conclude that since *abi-1* null mutants were not used in the analysis.

Most of our understanding of the function of SLI-1 comes from mammalian studies of its homolog c-Cbl in cell culture. These studies have demonstrated a large number of protein-protein interactions. To discover which of these interactions might be relevant to the engulfment inhibitory function of *sli-1,* we tested which domains were required to rescue SLI-1 function. The only essential domain was the N-terminal TKB domain. While our studies do not preclude a role for the C-terminal proline-rich or RING finger domains, they do indicate that these domains are not central to the engulfment and cell migration functions of SLI-1.

The TKB domain includes three motifs: a four helix bundle, a Ca^++^ binding EF hand and an SH2 domain. These three motifs together define a unique domain that binds phosphotyrosines of protein tyrosine kinases [26]. This binding, in turn, allows the E3 ubiquitin ligase function of the RING finger of Cbl to ubiquitinate and target these tyrosine kinases for destruction or sequestration. However, since the RING finger domain, which is required for ubiquitination, is partially dispensable for inhibition of cell migration by SLI-1, the above mechanism cannot explain our results.

In addition to tyrosine kinases, several other proteins have been shown to interact with the N-terminal TKB domain. One of them is APS, an adapter protein that is involved in insulin signaling [47]. However there is no obvious APS homolog in *C. elegans*. Furthermore, APS signaling requires the C-terminus of Cbl in mammals and the phenotypes we describe only partially require the C-terminal domain. Another interactor, SLAP, the Src-like adapter protein, also binds to the N-terminus of Cbl [48]. It, too, has no obvious homolog in *C. elegans*.

A third TKB domain interactor is tubulin. Alpha and beta tubulin bind to the Cbl N-terminus [49], [50], and Cbl co-purifies with tubulin in B-cell lysates [51]. The idea that an interaction between SLI-1 and tubulin is involved in engulfment suppression is intriguing for several reasons. First, it would support a role for microtubules in apoptotic cell engulfment, which until now has been shown to be regulated solely by actin cytoskeletal rearrangement. Second, it would fit with our genetic findings concerning *sli-1*. Specifically, *sli-1* inhibits both engulfment and DTC migration, two processes totally dependent on appropriate cytoskeletal regulation. Third, *sli-1* appears to act in parallel to all known engulfment genes and engulfment inhibitors. That, too, would be consistent with *sli-1* action affecting an entirely different molecular pathway, namely one regulating microtubules.

The discovery that *sli-1* acts through a pathway in parallel to the two core engulfment pathways (*ced-10* Rac and *ced-1*) suggests that there are still other cell biological processes involved in apoptotic cell engulfment yet to be discovered. Since the two core pathways were discovered over 20 years ago, it begs the question of why these processes were not identified previously. Possibly, defects in the unidentified processes result in embryonic lethality so they were not identified in genetic screens. Alternatively, these pathways are redundant with the core pathways and, therefore, would only be discovered in the absence of one or both of them. Regardless of the answer, the existence of other pathways suggests that very tight control of engulfment is required during development.

Much of the work on engulfment has been aimed at identifying which signals from the dying cell activate the *ced-10* Rac and *ced-1* pathways. Our findings suggest that in addition to the need for positive signals, engulfing cells require multiple inhibitory signals to prevent inappropriate engulfment. As discussed earlier, engulfment of dying cells promotes their programmed cell deaths. Potentially there are circumstances during development when cells are particularly susceptible to engulfment-mediated death, which, unless prevented, would result in excess cell death and developmental errors. Perhaps these inhibitory pathways exist as a failsafe mechanism to prevent such errors.

## Materials and Methods

### Strains and genetics

*C. elegans* strains were maintained at 22°C as described [52]. The N2 Bristol strain was used as the wild-type strain. Animals were grown on NGM plates and fed OP50 bacteria [4], [53]. The mutations and integrants used were: LGI: *ced-1(e1735, n2091), ced-12(n3261, tp2)*; LGIII: *abi-1(tm494), ced-6(n2095), ced-7(n1996)*; LGIV: *ced-2(n5101), ced-3(n2427), ced-5(n1812), ced-10(n1993), dpy-13(e184sd), let-60(n1046gf)*; LGV: *unc-76(e911), nIs96* [41]; LGX: *abl-1(ok171), nIs106* [41], *sli-1(n3538, sy143)*. Mutant alleles for which no citation is given were described previously [54]. Information about *ok* and *tm* alleles can be found at www.wormbase.org (*tm* alleles were kindly provided by S. Mitani, Tokyo Women's Medical University, Japan). The following balancer chromosomes were used: LGI; LGIII: *hT2[qIs48],* LGII: *mIn1[mIs14],* LGIV; LGV: *nT1[qIs51].*

We isolated *ced-2(n5101)* from a *C. elegans* deletion library; genomic DNA pools from the progeny of EMS or UV-TMP mutagenized animals were screened for deletions using PCR as described [55]. *ced-2(n5101)* removes 637 nucleotides from chromosome IV, 242 base pairs 5′ to the *ced-2* ATG, the entire first exon (439 bp) and 12 bp of the first intron.

### Quantitation of engulfment defects

Unengulfed apoptotic corpses were visualized in the heads of young larvae as refractile discs directly using Nomarski differential interference contrast (DIC) microscopy [56], [57]. Apoptotic cell corpses were counted in the heads of first larval stage (L1) animals within 30 min of hatching, except for animals treated with RNAi (see below). Animals were anaesthetized in 30 mM sodium azide in M9 [53] and viewed using DIC optics on a Zeiss Inverted Axio Observer compound microscope (Thornwood, NY, USA). For animals treated with feeding RNAi, L1 animals were picked, and those with gonads that had not passed the 4-cell stage (all within 60 minutes of hatching) were viewed as described above. *p* values for pairwise comparisons were calculated using the Student's t test.

### Quantitation of cell-death defects

For quantitation of cell-death defects in the anterior pharynx, animals in the third larval stage (L3) were anaesthetized and viewed with DIC microscopy as described above. Briefly, the locations of the nuclei of the 16 cells that undergo programmed cell death in the anterior pharynx are known [39]. In wild-type animals by the L3 stage, all of those nuclei have disappeared; any nuclei in these locations in the animals examined at the L3 stage were scored as extra cells. *p* values for pairwise comparisons in the pharynges were calculated using Student's t test.

### Time-lapse microscopy

Single embryos were placed on agar pads, sealed with petroleum jelly and viewed at 20°C using a Zeiss Inverted Axio Observer compound microscope equipped with Nomarski DIC accessories, a Zeiss AxioCam HRm digital camera and Zeiss Axiovision image acquisition software. Pictures were taken every 3 min for 200 min, and images were analyzed beginning with the appearance of the first cell corpse and ending at the comma stage. The time of appearance of each corpse was recorded. For each time point, 60–80 serial z sections at 0.4 µm/section were recorded. Images were analyzed with ImageJ64 1.45 s (http://imagej.nih.gov/ij) using the plugin Cell Counter. *p* values for comparisons between strains were calculated using the Wilcoxon rank-sum test.

### Quantitation of DTC defects

Adult animals 18 h after the mid-fourth larval stage (L4) were anaesthetized and viewed as described above in Quantitation of engulfment defects and gonads were visualized [44], [58]. Only gonads that were completely visualized were scored. Specifically, gonads that were partially occluded by other structures were not scored. DTC migration was scored as defective when the gonad was morphologically abnormal (extra turn, two arms or bizarre twists) or when the gonad was short or long. Gonadal length was defined as abnormal when the gonad tip was distal to the ipsilateral spermatheca (short) or distal to the contralateral spermatheca (long). The vast majority of abnormalities were in morphology rather than in length. *p* values for pairwise comparisons were calculated using Fisher's exact test.

### Expression analysis of *sli-1*

For the transcriptional GFP fusion, a PCR product encoding the 5 kb genomic fragment upstream of the M02A10.3a (*sli-1*) start site was made with SalI/XbaI ends. The product was then digested with SalI and XbaI and ligated to pPD95.75 from the Fire Lab *C. elegans* kit (Addgene). The resulting plasmid contained the 5 kb upstream of M02A10.3a adjacent to *gfp* (GFP[S65C]). The plasmid was injected into gonads of N2 animals with the coinjection marker P*_unc-122_::rfp* (50 ng/µl for each with 50 ng/µl 1 Kb Plus DNA Ladder (Invitrogen) to a total concentration 150 ng/µl). Three independent transgenic lines were observed and photographed using fluorescence and DIC microscopy. For the translational GFP fusion, we used *in vivo* recombination (http://wormbook.org/chapters/www_reportergenefusions/reportergenefusions.html). Fosmid WRM0611cB12 was digested with MscI and SpeI, generating a 9 kb fragment which includes 5 kb of sequence upstream of M02A10.3a and 4 kb of the M02A10.3a sequence. To make the second fragment, a 5 kb full length M02A10.3a sequence was PCR amplified from fosmid WRM0611cB12 and then inserted into vector pDEST-MB14 using the Gateway method (Invitrogen), resulting in an in-frame fusion of M02A10.3a with GFP at its C-terminus. Then this plasmid was cut with PstI and SacII, making a 6 kb fragment including the C-terminal 4.5 kb of M02A10.3a fused with *gfp* and some additional sequence from pDEST-MB14. The 2 fragments were mixed with the co-injection marker P*_myo-2_::rfp* (50 ng/µl for each with 50 ng/µl 1 Kb Plus DNA Ladder to a total concentration 200 ng/µl) and injected into the gonads of *ced-10(n1993);sli-1(sy143)* animals. Three independent transgenic lines were analyzed. All lines demonstrated rescue of the *sli-1* engulfment suppression defect.

### *sli-1* rescue

P*_hsp_sli-1wt,* P*_hsp_sli-1ΔN,* P*_hsp_sli-1ΔRING,* and P*_hsp_sli-1ΔC* were described previously [30]. Briefly, *sli-1ΔN* encodes the first 64 amino acids of SLI-1 followed by a short linker (Leu Ala Leu) and then amino acid (aa) 350 through the end of the protein (aa 583). *sli-1ΔRING* encodes the first 393 amino acids followed by the following linker (Glu Thr Gly Thr Thr Phe Glu) and then amino acid 432 through the end of the protein. *sli-1ΔC* encodes the first 447 amino acids of the protein. The P*_hsp_gfp* plasmids have been described previously [59]. Each minigene was expressed under the control of the *hsp16/2* and *hsp16/41* promoters. P*_hsp_* plasmids were injected into *ced-10(n1993); sli-1(sy143)* animals at a concentration of 20 ng/µl with a plasmid containing *myo-2::rfp* as a coinjection marker at 5 ng/µl and with 35 ng/µl of 1 Kb Plus DNA Ladder for a total concentration of 80 ng/µl per injection. The pharynges of transgenic animals were RFP-positive. For quantification of unengulfed apoptotic cell corpses, embryos were grown at 20°C, heat-shocked for one hour at 33°C, placed at 20°C for up to four hours after which cell corpses in the heads of newly hatched first larval stage (L1) animals were counted. For quantification of DTC migration defects, animals were heat shocked for one hour at 33°C and placed at 22°C for 30 hours. DTC morphology in young adults was then analyzed in an equal number of animals with and without the transgenic arrays. 200 gonad arms were analyzed per genotype. Two independent transgenic lines were analyzed for each transgene combination except for the engulfment analysis of P*_hsp_sli-1ΔRING,* in which only one line was used. This was because only one of the lines produced viable L1 larvae after heat shock during embryogenesis. Attempts were made with five separate lines. We presume this line had lower expression levels based on the fact that high expression levels of SLI-1 proteins are toxic to worms. Also, the line that produced viable larvae had comparatively faint GFP staining.

### RNA interference by feeding

Animals were fed bacteria that contained either the RNAi empty feeding vector L4440 [60] or an RNAi feeding vector with part of the *abi-1* gene, *B0336.6*, cloned into it. We obtained the *abi-1* feeding plasmid from Open Biosystems (Huntsville, AL, USA). The DNA sequence of the clone was determined to verify its accuracy. Feeding RNAi was performed as described [60], [61]. Briefly, bacteria were grown in liquid culture overnight and then transferred to NGM plates containing 1 mM isopropyl-D-β-thiogalactopyranoside (IPTG). Fourth-larval stage (L4) animals were placed on these plates and 24 h later were transferred to fresh plates. Progeny were tested for engulfment or DTC migration defects.

## Supporting Information

Figure S1 Expression pattern of *P_sli-1_::gfp. gfp* was expressed under control of the *sli-1* promoter. i, iii, v, vii and ix show fluorescence images and ii, iv, vi, viii and x show accompanying DIC images. i and ii, embryo at gastrulation; iii and iv, embryo at 1½-fold stage; v and vi, L1 head; vii and viii, L1 body; ix and x, L4 gonad with arrowheads showing DTC. Bar = 5 microns.
(TIF)

Table S1 Overexpression of *sli-1* might cause an engulfment defect in twofold embryos. Twofold embryos were viewed using DIC microscopy. The numbers of cell corpses were counted. s.d., standard deviation.
(DOCX)
