# Supplementary figures and images for: SLI-1 Cbl Inhibits the Engulfment of Apoptotic Cells in C. elegans through a Ligase-Independent Function
Source: PLoS Genet. 2012 Dec 13;8(12):e1003115. doi: 10.1371/journal.pgen.1003115 (PMC3521709; doi:10.1371/journal.pgen.1003115)

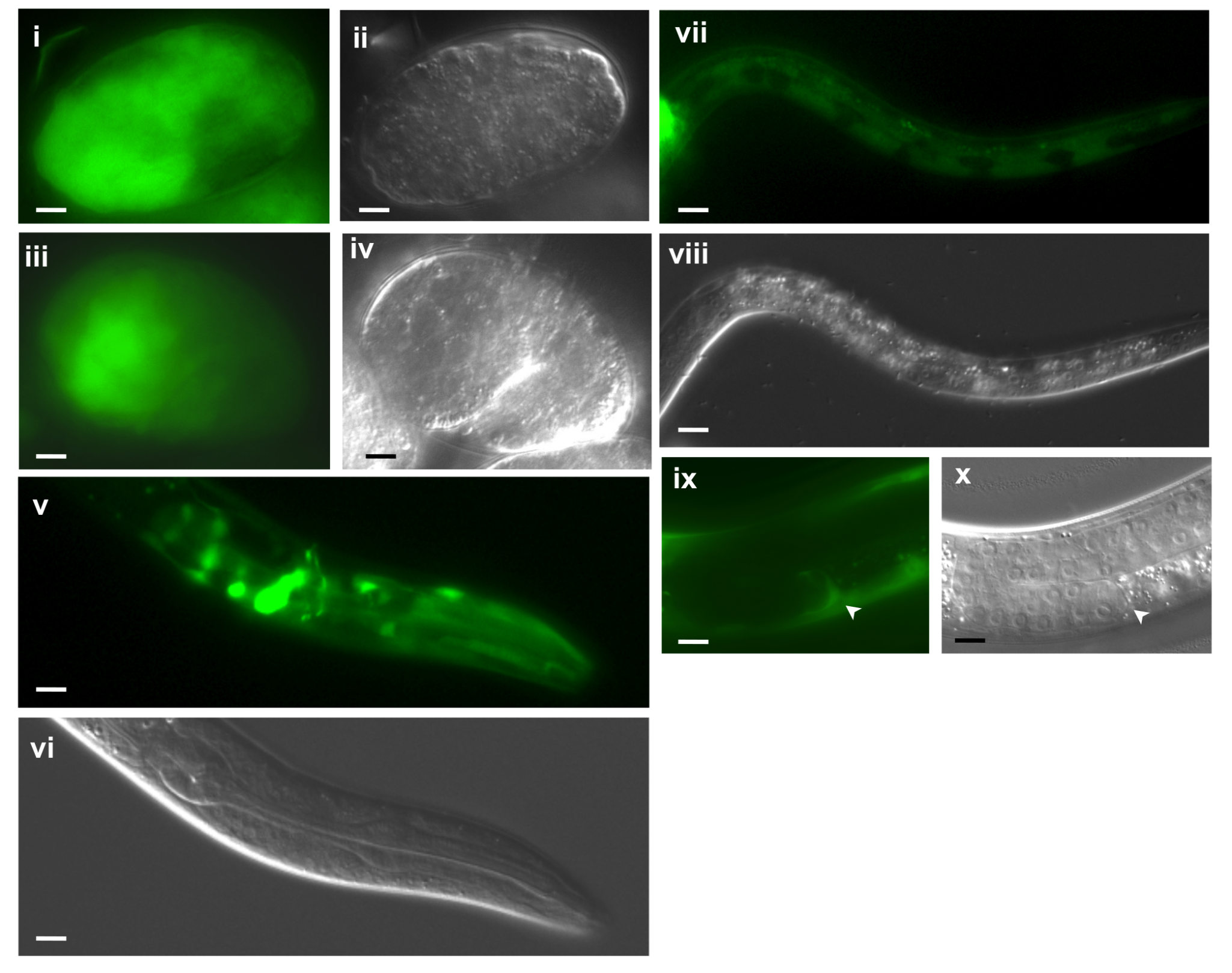

Supplement: Figure S1 — Expression pattern of Psli-1::gfp. gfp was expressed under control of the sli-1 promoter. i, iii, v, vii and ix show fluorescence images and ii, iv, vi, viii and x show accompanying DIC images. i and ii, embryo at gastrulation; iii and iv, embryo at 1½-fold stage; v and vi, L1 head; vii and viii, L1 body; ix and x, L4 gonad with arrowheads showing DTC. Bar = 5 microns. (TIF) [file pgen.1003115.s001.tif]
