# Supplementary material for: SLI-1 Cbl Inhibits the Engulfment of Apoptotic Cells in C. elegans through a Ligase-Independent Function
Source: PLoS Genet. 2012 Dec 13;8(12):e1003115. doi: 10.1371/journal.pgen.1003115 (PMC3521709; doi:10.1371/journal.pgen.1003115)
Supplement: Table S1 — Overexpression of sli-1 might cause an engulfment defect in twofold embryos. Twofold embryos were viewed using DIC microscopy. The numbers of cell corpses were counted. s.d., standard deviation. (DOCX) [file pgen.1003115.s002.docx]

**Supplemental Information**

**Table S1. Overexpression of *sli-1* might cause an engulfment defect in twofold embryos**

| **Transgene** | **Corpses ± s.d.** | **n** | ***p*-Value** |
| --- | --- | --- | --- |
| none | 8.1 ± 1.3 | 15 | <0.04 |
| P*_sli-1_sli-1::gfp* | 9.2 ± 1.6 | 17 |  |

Twofold embryos were viewed using DIC microscopy. The numbers of cell corpses were counted. s.d., standard deviation.
